# ‘Use a Thorn to Draw Thorn’ Replacement Therapy for Prevention of Dental Caries

**DOI:** 10.5005/jp-journals-10005-1068

**Published:** 2010-09-15

**Authors:** Seema Gupta, Nikhil Marwah

**Affiliations:** 1 Senior Resident, Department of Pedodontics and Preventive Dentistry, Maulana Azad Institute of Dental Sciences, New Delhi, India; 2 Reader, Department of Pediatric Dentistry, Mahatma Gandhi Dental College, Jaipur, Rajasthan, India

**Keywords:** Replacement therapy, Dental caries prevention, *Streptococcus mutans.*

## Abstract

Despite the use of conventional physical and chemotherapeutic agents for caries management, dental caries still continues to be the most prevalent oral infectious disease. Thus, there is a need of additional caries prevention approaches. Strain replacement therapy is one such novel approach. In this, relatively avirulent strains of *Streptococcus mutans* produced by recombinant DNA technology are implanted into the oral cavity. These may either interfere with the colonization of, or compete with the indigenous cariogenic mutans streptococci. This technique might provide a cost-effective, long-term means of achieving tailor made protection for the host against dental caries.

## INTRODUCTION

Oral cavity is a complex ecosystem in which a rich and diverse microbiota is present. Dental caries and periodontal diseases occur in nearly 95% of general public. Considerable evidence has implicated a particular bacterium *S. mutans* as the principal causative agent of dental caries. This may be due to its ability to stick to the tooth surface,^1^ produce large amounts of acid from metabolism of dietary sugars,^2^ and production from sucrose of extracellular polysaccharides that trap the acid so produced.^3^

Current methods of caries management include traditional prevention (prophylaxis, fluorides), early surgical intervention/minimal intervention, lesion monitoring and recall, and finally, delayed intervention.^4^

## NEED OF A NEW APPROACH FOR DENTAL CARIES: REPLACEMENT THERAPY

Despite the use of conventional physical and chemo-therapeutic agents for caries management, dental caries still continues to be the most prevalent oral infectious disease. Clearly, additional caries prevention approaches, which can augment the existing ones (e.g. fluorides, brushing, flossing, etc), are highly desirable.

## PRINCIPLE OF REPLACEMENT THERAPY

There are many positive and negative interactions among different species of bacteria inhabiting the oral ecosystem. The ability of a given bacterium to colonize a host is determined by a number of factors, such as the bacterium’s metabolic needs, and the interactions of the bacterium with the pre-existing bacterial flora. Bacterial interactions can be generally classified as either “positive” or “negative.” In positive interactions, an “effector” bacterial strain alters the microenvironment to promote colonization of a second, “target” organism. In “negative” interactions, the effector strain alters the microenvironment in a manner that decreases or completely prevents target bacterium colonization. Negative interactions between competing bacteria during host colonization are commonly termed bacterial interference.^5^

Humans have long attempted to intervene in these bacterial interactions. This has led to the development of a novel therapeutic approach of preventing microbial diseases (like dental caries) called whole bacteria replacement therapy (currently called as probiotic therapy).^6^ Ingestion of probiotic bacteria, particularly lactobacilli is commonly practiced to promote a well-balanced intestinal flora.^7^

Florey (1946) pointed out that the use of beneficial bacteria to fight pathogenic bacteria has been attempted by many laboratories for many decades. This idea, traditionally called as replacement therapy, is very appealing from both practical and theoretical standpoints. The development of antibiotics revolutionized the practice of medicine in the second half of 20th century. Since 1982, however deaths stemming from infectious diseases have steadily climbed in parallel with the rise of antibiotic resistant pathogens. A wide variety of medically important bacteria is becoming increasingly resistant to commonly used antibiotics. The fear is that we will, in effect, return to preantibiotic era unless new antibiotics or new approaches, such as replacement therapy are developed soon.

In case of dental caries, we can confidently assume that evolution will continue to act on principal etiological agents, mutans streptococci to bring them into a new climax state wherein they no longer express a pathogenic potential. However, for natural selection to act via spontaneous mutation to eliminate this organism’s natural virulence, thousands of years may be required to complete. The intention of modern day replacement therapy, as it applies to diseases caused by indigenous microorganisms, is to greatly speed this natural evolutionary process. By careful study of the pathogen, it may be possible to identify and modify certain of its genes to create a so-called effector strain in a relatively short span of time that presages the climax organism naturally selected by evolution.^8^

Majority of effort in the field of replacement therapy to prevent of dental caries has centered on isolating effector strains with decreased acidogenic potential. This is a microorganism that does not cause the disease itself, but rather persistently colonizes host tissues, which are susceptible to infection by a particular pathogen. By virtue of its presence, it must somehow be able to prevent infection by that pathogen whenever the host is exposed to it.^2^ A “recombinant Streptococcus mutans strain” is a non-naturally occurring strain of *S. mutans* that has been generated using any of a variety of recombinant nucleic acid techniques (i.e. techniques involving the manipulation of DNA or RNA).^5^

However, to prevent dental caries, an effector strain must have following prerequisites:

1. It must have a significantly reduced pathogenic potential to promote caries.
2. It must persistently colonize *S. mutans* sites, thereby preventing colonization by disease causing strains whenever host comes in contact with them.
3. It must aggressively displace indigenous strains of *S. mutans* and allow previously infected subjects to be treated with replacement therapy.
4. It must be safe and not make the host susceptible to other disease conditions.^1^

## TYPES

There are two types of strain replacement therapy:

1. Pre-emptive colonization
2. Competitive displacement

1. In pre-emptive colonization studies, *S. mutans,* which were unable to produce caries either due to their inability to produce lactic acid (lactate dehydrogenase mutants) or to synthesize intracellular polysaccharides (ICP mutants) were implanted into the oral microflora of experimental animals prior to the introduction of potentially pathogenic strains of *S. mutans.^9^* The concept is that the nonvirulent *S. mutans* will have an ecological niche similar to that of virulent *S. mutans,* thus will be capable of interfering with colonization by the cariogenic bacteria. The time in an infant’s life when *S. mutans* first colonize the teeth is the ideal period in which to implant effector strains.^10^
2. In competitive displacement, a noncariogenic microorganism is introduced that is capable of competing with and displacing the indigenous cariogenic MS. An example of such a strain is *S. salivarius* TOVE-R (a rough colony variant of strain TOVE-S), which preferentially colonizes the tooth surfaces rather than the tongue. Strain TOVE-R was shown capable of growing faster than MS^11^ and when given orally to rats, it soon became prominent in the animals’ dental plaque and brought about a reduction in the levels of MS and dental caries.^12^ The ideal effector strain would be a noncariogenic bacterium, which is continuously present in the mouth and competes successfully with MS. It should accumulate preferentially on the tooth surfaces, be able to grow rapidly and withstand sudden and wide changes of pH.^13^

## STRATEGIES

1. One of the physiological properties, which might provide a strong selective advantage in colonization is bacteriocin production, known for sometime to be a common feature of *S. mutans.* Bacteriocins are proteins that kill representatives of same species as the producer organism or related species.^14^ In a preliminary screening of *S. mutans* strains for bacteriocin production, one strain, *JH1001* inhibited the growth of virtually every other strain of this organism.^15^ The exact chemical nature of the inhibitory substance produced by *JH1001* has not yet been determined. Preliminary studies indicate that it is a small molecule (molecular weight approximately 1000) that is synthesized in detectable amounts only during stationary phase.^16^ In another study, strain *JH1001* was implanted onto the teeth of five human volunteers harboring high levels of MS. The ability of strain *JH1001* to superinfect and persistently colonize the human oral cavity was tested. It was found that two and half years later, *JH1001* was still found to be present in three subjects and in one of these, no indigenous MS could be detected.^17^
2. Colonization of the human oral cavity by the mutant of *JH1001* that produces three-fold elevated inhibitor activity has also been tested. In this case, a single five-minute infection regimen that involved brushing and flossing approximately 10^11^ cells onto the teeth resulted in persistent colonization of all three subjects tested. In two of these subjects, significant (7- and 38-fold) reductions in the numbers of their total *S. mutans* had been recorded. No changes in the levels of other plaque bacteria, such as *S. sanguis* had been observed. The results of this study indicated that a practical and effective regimen for the implantation of an effector strain could be developed for the replacement therapy of dental caries in humans.^18^
3. Mutants of *S. mutans* defective in intracellular polysaccharide metabolism have also received attention. Studies by Tanzer et al have indicated the ability of IPS mutants of *S. mutans* to colonize the teeth of experimental animals pre-emptively.^19^
4. A natural variant of *S. salivarius* called *TOVE-R* has been studied. Like typical *S. salivarius* strains, *TOVE-R* is noncariogenic.^20^ Atypically, it preferentially colonizes tooth surfaces and produces plaque much like *S. mutans.^12^*The basis for the ability of *TOVE-R* to compete successfully with mutans streptococci is currently not known, but may be related to its ability to grow faster *in vitro* over a range of experimental conditions.^11^
5. Lactic acid is the strongest of the metabolic acid end products of oral microorganisms, and its production is catalyzed by lactate dehydrogenase (LDH). Hillman hypothesized that LDH^-^ mutans streptococci would have reduced cariogenic potential and that they could be useful effector strains for replacement therapy of dental caries.^21^ However, at high sugar concentrations, the levels of activity of these enzymes are apparently insufficient to compensate for the absence of LDH. A supplemental alcohol dehydrogenase (ADH) activity can complement the LDH deficiency when expressed in the temperature sensitive LDH mutant.^22^

LDH-deficient mutants of *S. mutans* produced approximately half as much titrable acid as did their parent when grown in a broth containing excess glucose.^21^ Germ-free rats infected with an LDH-deficient mutant and fed a high sucrose diet for 14 weeks were found to have a 90% lower incidence and severity of caries lesions, compared with those of animals infected with the parent strain. The mutant was found to colonize the teeth of animals to the same extent as did its parent. The difference in cariogenic potential between parent and mutant, therefore, could be ascribed solely to differences in acid production.^23^

Strain *JH1140* is a derivative of an *S. mutans* strain isolated from the saliva of a human subject. It naturally produces an antibiotic called mutacin 1140 that is capable of killing all others strains of *S. mutans* tested to date, which gives it a selective advantage in colonization as demonstrated in both animal models^24^ and humans.^25^ Recombinant DNA methods were used to delete essentially the entire open reading frame (ORF) for lactic acid dehydrogenase. This mutation created a metabolic blockade that was lethal when exchanged for the wild-type allele, but it was found that replacing the ORF for LDH with the ORF for alcohol dehydrogenase B from Zymomonas mobilis overcame this blockade to yield a viable strain called *BCS3-L1* that produced wild-type levels of mutacin 1140.^26^ Also, fermentation end-product analysis revealed that *BCS3-L1* produced no detectable lactic acid.^27^ As predicted from earlier work, most of the metabolized carbon was converted to neutral end products, ethanol and acetoin.^28^ Under various cultivation conditions, including growth on a variety of sugars and polyols, such as sucrose, fructose, lactose, mannitol and sorbitol, *BCS3-L1* yielded final pH values that were 0.4 to 1.2 pH units higher than those of its parent, *JH1140^21^*

The ability of *BCS3-L1* to serve as an effector strain in the replacement therapy of dental caries was extensively tested in the laboratory and animal models.^27^ The strain proved to have significantly reduced pathogenic potential. It persistently and pre-emptively colonized the niche on the tooth surface normally occupied by wild-type strains of *S. mutans.* It was genetically stable and showed no ill effects in acute or chronic toxicity studies.^4^ No gross or microscopic abnormalities of major organs were associated with oral colonization of rats with *BCS3-L1* for a period of six months.^27^ Sufficient mutacin 1140 has not been purified to directly test its toxicity. However, the prototype lantibiotic, nisin, is known to have extremely low toxicity, and has been developed and used for decades as a food preservative that is generally recognized as safe.^29,30^

It is conceivable that mutacin production by *BCS3-L1* and the fermentation products resulting from LDH deficiency could alter plaque ecology and produce another microorganism with pathogenic potential. However, the mutacin 1140 producing strain of *S. mutans* eliminated mutacin-sensitive indigenous strains of *S. mutans* but had no effect on indigenous *S. oralis* strains that were equally sensitive to mutacin killing *in vitro.* These results indicate that *S. mutans* has a physically distinct habitat that is separated from the *S. oralis* habitat by a distance sufficient for dilution to reduce the concentration of mutacin below its minimal inhibitory concentration.^25^

If *BCS3-L1* could be shown to have similar properties in humans, it would serve as an idealized effector strain with the following advantages:^4^

- A single treatment regimen could, ideally, provide lifelong protection against tooth decay.
- The possibility of deleterious side effects are negligible as the effector strain is essentially identical to a microorganism, which is found universally in humans.
- Minimal patient education and compliance is required.
- The therapeutic method would be cost-effective and suitable for use in the population at large, and would be particularly well suited for use in developing countries.

6.   J.D. Hillman and others studied the ability of daily applications of *Streptococcus rattus strain JH145* to affect the numbers of an implanted *Streptococcus mutans* strain in a rat model. A spontaneous L(+)-lactate dehydrogenase (LDH)-deficient mutant of Streptococcus rattus, *JH146,* was isolated by screening on selective medium and compared with a previously isolated spontaneous LDH deficient strain, *JH145.* Both strains were shown to have single base pair deletion mutations in the structural gene (LDH) for LDH, and reversion frequencies were approximately the same. Animals treated once daily with ≥ 10^6^ CFU (colony forming units) of *JH145* showed a statistically significant decrease in the proportion of implanted *S. mutans* to total cultivable bacteria in oral swab samples. The rate of decrease in *S. mutans* levels was dose-dependent. No adverse effects were observed by in life observation of treated animals, and histopathological, hematological and blood chemistry analyses were unremarkable. The results indicated that daily application of *JH145,* a naturally occurring LDH-deficient variant of *S. rattus,* can compete with *S. mutans* for its habitat on the tooth surface and *S. rattus JH145* has potential as a probiotic for use in the prevention of dental caries.^31^
7.   Genetically modified probiotics with enhanced properties can be developed (‘designer probiotics’). For example, a recombinant strain of Lactobacillus that expressed antibodies targeting one of the major adhesions of *S. mutans* (antigen I/II) was able to reduce both the viable counts of *S. mutans* and the caries score in a rat model.^32^

Clinical studies have indicated that bacteria with established probiotic effects (lactobacilli and bifidobacteria) have some promise for prevention of caries. Lactobacillus rhamnosus GG ingested in dairy products (milk, cheese) reduced salivary mutans streptococcal counts in adults and protected against caries in children.^33,34^ Other lactobacilli have also been shown to reduce mutans streptococcal counts in saliva. Lactobacillus reuteri, when delivered by yoghurt,^35^ straw or tablet,^36^ chewing gum^37^ or as a lozenge,^38^ significantly reduced the counts of mutans streptococci in saliva (p < 0.05). The short-term consumption of yoghurt^39^ or ice cream^40^ containing Bifidobacterium spp. resulted in a significant reduction in salivary mutans streptococci (p < 0.05) but not in lactobacilli. Other studies have reported reductions in mutans streptococci levels in saliva following the use of probiotic containing yoghurts.^41^

8.   Caries development is usually a prolonged process involving cycles of demineralization and remineralization. Periods of plaque acidification and tooth demineralization are normally followed by phases of alkalinization with a return to more neutral plaque pH values,^42^ which promotes remineralization at the tooth surface.^43,44^ It is when the phases of demineralization dominate that a carious lesion develops. Whereas the processes by which dental plaque becomes acidified have been intensively studied, the alkalinization phase and pH homeostasis during fasting periods are rather poorly understood. A number of mechanisms is thought to contribute to the alkalinization of dental plaque, including clearance of acids and sugars by saliva, buffering by salivary and bacterial components, and production of alkali by plaque bacteria, which occurs primarily through the metabolism of urea to ammonia by microbial urease activity.^45^

Urea is secreted continuously in the range of 3 to 10 mM in saliva and crevicular fluids of healthy individuals^46^ and rapidly hydrolyzed by the urease enzymes of oral microflora. Existing data indirectly supports a major role for ureolysis in plaque pH homeostasis. Elevated salivary urea and ammonia concentrations are correlated with marked reductions in the extent and duration of plaque acidification following a carbohydrate challenge.^47^ Chen YYM and others indicated that physiologically relevant levels of urea (2 to 10 mM) can dramatically alter environmental acidification even in the presence of a significant glucose excess.^48^ Urea hydrolysis can neutralize plaque acids^49^ and may positively influence plaque ecology by preventing the pH from falling to levels that select for the outgrowth of aciduric, cariogenic microorganisms.^50^ In addition, ammonia released by ureolysis can promote remineralization of the tooth enamel.^43^

Clinical studies indicate that caries-resistant patients have elevated resting plaque pH values and that these values are not lowered to the same extent as those in caries-susceptible individuals following a carbohydrate intake.^51^ Margolis et al have confirmed that caries-resistant subjects have more alkaline resting plaque pH values than do caries-susceptible individuals and have correlated this, in part, with increased ammonium concentrations in plaque.^43^ Studies of patients with chronic renal failure have also shown that these patients, who have salivary urea concentrations often greater than 50 mM, have alkaline plaque pH levels and a very low incidence of dental caries, despite ingestion of a diet dominated by carbohydrates.^52^

Recently, attention has focused on the concept that the development of cariogenic plaque may result not only from the extensive acidification of dental plaque but also from a diminution in the alkali-generating capacity of oral biofilms colonizing a carious lesion.^53^ Loss of ammonia-producing bacteria from the complex populations on the tooth surface would reduce the capacity of plaque to neutralize acids and slow the return of plaque pH to more neutral values. This concept is consistent with two observations: (i) the resting plaque pH in healthy individuals is higher than the pH of the saliva which bathes the plaque, (ii) the depth and duration of plaque acidification is greater in caries-prone subjects. Also reinforcing this concept is the demonstration that individuals with low salivary ureolytic capacity have a markedly diminished capacity to blunt glycolytic acidification.^54^ Most recently, recombinant *Streptococcus mutans* strains carrying plasmid-borne urease genes were used *in vitro* to demonstrate directly that the levels of urease commonly found in healthy plaque are sufficient to offset a pH drop by using physiologically relevant levels of urea, even in the presence of a 10-fold molar excess of glucose.^55^ Importantly, the ureolytic capacity of dental plaque within a carious lesion and the prevalence of alkali-generating bacteria following sustained acidification of dental plaque remain unexplored.^45^

Unfortunately, testing of hypotheses related to the base-producing capacity of biofilms and oral health in well-controlled clinical studies has yet to be undertaken. A variety of accepted animal caries models is available for testing the effects of various carbohydrates and therapeutics on caries formation, with the rat model appearing to be the most appropriate and widely accepted. However, these caries models are not readily adaptable to the study of alkali-generation because of the lack of a suitable test organism and differences in the endogenous flora of rats and humans with regard to urease activity.^45^ In clinical studies, essentially all of the data relating ureolysis to plaque pH homeostasis and oral health are restricted to total plaque from healthy individuals.^49^ To address some of these deficiencies, recombinant ureolytic strains of the cariogenic plaque bacterium *S. mutans,* which could be implanted in the plaque of experimental animals were constructed to test the hypothesis that enhancing the alkali-generating capacity of plaque could reduce the incidence of caries formation.^45^

Increasing base production in dental plaque reduces not only smooth surface caries but also sulcal scores. Sulcal surfaces are areas where the diet and acidic breakdown products are likely to be retained, more than on the smooth surfaces of the teeth, therefore the sulci are generally subjected to a more severe acid attack and more prone to caries development. Clinically, sulcal and interproximal caries are far more common than smooth surface caries and fluoride is far less effective against these types of caries. Therefore, it appears that in contrast to some other caries prevention strategies, enhancement of ammonia generation in plaque might be an effective way to combat the more common types of carious lesions.^45^ The ability to use genetically engineered, base-producing oral bacteria in controlled experiments should provide valuable information, which may also lead to the development of strains that could eventually prove useful for control of dental caries by replacement therapy.^48^

One clear advantage that ammonia-producing plaque bacteria have over other approaches is that they may favorably modify the supragingival plaque ecology by fostering an environment that inhibits the emergence of cariogenic flora rather than targeting a specific pathogenic agent or perturbing the normal metabolic or physiologic pathways, which could compromise competitive fitness.^45^

A key consideration in the utility of replacement strains is their ability to compete with endogenous strains and the fact that ablation of endogenous activities or introduction of foreign genes can compromise the fitness of a bacterium. Arguably, the introduction of genes producing ammonia from urea into oral streptococci may instead create strains, which are better able to compete than the parent. First, urea can diffuse through the membrane so that no energy is required to transport this compound. Once cleaved, the ammonia can neutralize the cytoplasm raising the intracellular pH and creating a diminished requirement for the organisms to spend ATP to extrude protons. Recently, it has also found that the ureolytic oral bacteria *A. naeslundii* can use the ammonia from urea efficiently as a source of nitrogen. Thus, recombinant ureolytic bacteria may gain a competitive advantage from a bioenergetic standpoint because they can access a source of nitrogen unavailable to other oral bacteria.^56^ Modulation of the alkali-generating potential of dental plaque may also have great potential because it does not target particular etiologic agents and instead may work by fostering an ecologically healthy oral environment, which naturally controls the emergence of pathogenic microorganisms.^45^

9.   A variety of bacteria including *S. sanguis* is able to utilize arginine catabolically via argnine deaminase system.^57,58^ In this pathway, AD catalyses the hydrolysis of arginine to citrulline and ammonia.^58^ Though *S. sanguis* is inherently less acid tolerant than other organisms like *S. mutans,* it can be protected against lethal acidification by catabolism of arginine by AD pathway. Protection probably occurs through production of ammonia and associated rise in environmental pH.^59^ This protection may be critical to the survival of *S. sanguis* in dental plaque in which pH value can drop below 4.0 and in which cycles of acidification-alkalinization occur frequently.^60^

Thus, with the isolation of genes from *S. sanguis,* it should be possible to introduce the AD system into cariogenic bacteria *S. mutans.^61,62^* These recombinant organisms should have a selective advantage because of their ability to produce 1 molecule of ATP per molecule of arginine catabolized. The ammonia producing bacteria should not only be less cariogenic themselves, but also could decrease the overall cariogenic potential of dental plaque.^60^ Studies have demonstrated the arginine and arginine-containing compounds are far more effective than other amino-acids or peptides at inhibiting pH drop following a sugar challenge and in eliciting a subsequent pH rise.^63^ Thus, ammonia production from arginine by oral streptococci like *S. sanguis* and *S. mitior* could play a direct, ameliorative role in initiation and progression of dental caries.^60^

## BACTERIA TESTED AS POTENTIAL ‘EFFECTORS’ FOR REPLACEMENT THERAPY

Effector species Inhibitory agent Investigator:

- *S. mutans* JH1000 and derivatives Mutacin 1140 Hillman^15,17,27^
- *L. rhamnosus* GG Not defined Nase^33^
- *S. equi subsp. zooepidemicus* Zoocin A Simmonds^64^
- *S. salivarius* TOVE-R Not defined Kurasz^11^
- *E. faecalis* Bacteriocin Gilmore.^65^

## ADMINISTRATION

The recombinant *S. mutans* strains of the invention can be orally administered in a variety of ways. For example, the recombinant *S. mutans* strains may be administered in the form of suspensions, chewable tablets, pills, capsules, sustained release formulas (e.g. an oral implant containing the recombinant *S. mutans* strain) or lyophil powders. The recombinant *S. mutans* strains can also be administered by direct application of a lyophil, culture, or cell paste to the teeth of the patient. Any mode of administration is suitable as long as the therapeutic composition is applied to the oral cavity. Preferably, the recombinant *S. mutans* is administered by applying a bacterial cell suspension directly to the teeth of the patient, e.g. by brushing and flossing.^5^

In general, the amount of recombinant *S. mutans* administered to the patient will be an amount effective for replacement of dental caries-causing *S. mutans* strains in the oral cavity of the host. “An amount effective for the replacement of dental caries-causing *S. mutans* strains in the oral cavity of the host” means an amount effective for oral cavity colonization by the recombinant *S. mutans* strain, and elimination of the resident, lactic acid-producing, dental caries-causing *S. mutans* strains (e.g. by competition between the bacteria for nutrients and/or by the production of a bacteriocin by the recombinant *S. mutans* strain). The term “unit dose” when used in reference to a pharmaceutical composition of the present invention refers to physically discrete units suitable as unitary dosage for the subject, each unit containing a predetermined quantity of active material (e.g. viable recombinant *S. mutans)* calculated to produce the desired therapeutic effect in association with the required diluent; i.e. carrier, or vehicle.^5^

Specific dosages can vary widely according to various patient variables, including size, weight, age, disease severity (e.g. the tenacity and/or number of lactic acid-producing, dental caries-causing resident *S. mutans)* and responsiveness to therapy (e.g. the susceptibility of the host’s oral cavity to colonization). Methods for determining the appropriate route of administration and dosage are generally determined on a case-by-case basis by the attending dentist or other clinician.^5^

In general, the number of recombinant *S. mutans* administered to the patient will range from about 10^2^ to 10^15^ bacteria, preferably from about 10^3^ to 10^14^ bacteria, more preferably from about 10^5^ to 10^12^ bacteria, normally about 10^11^ bacteria. Dosages appropriate for administration can be readily extrapolated from dosages sufficient for Streptococcus spp. colonization of the oral cavity in an animal model, e.g. *S. rattus* colonization of the oral cavity of rats.^5^ Appropriate dosages can also be estimated from dosages found appropriate for colonization of the human oral cavity by the bacteriocin-producing strain *S. mutans* JH1000, and variants thereof that express varying levels of bacteriocin activity.^25^

Multiple doses of the recombinant *S. mutans* strains can be administered to achieve oral cavity colonization and replacement of the resident, dental caries-causing *S. mutans* strains of the host. In general, the recombinant *S. mutans* strains of the invention need only be administered to the patient one time. Where the recombinant *S. mutans* strain is an auxotroph, colonization of the strain must be maintained by administration of a composition containing the appropriate organic substance. For example, where the recombinant *S. mutans* strain is a D-alanine auxotroph, D-alanine must be administered for persistent oral cavity colonization. D-alanine, or other appropriate organic substrate, is generally administered at least once a week, preferably at least once a day, more preferably at least twice a day, and can be administered as part of the patient’s routine dental care, e.g. as a component of a toothpaste, floss, or mouthwash.^5^

## NEWER APPROACHES

1. Production of Auxotrophic, Recombinant *S. Mutans* Strains: In the event that it becomes desirable to rid the host of the recombinant *S. mutans* colonizing the oral cavity, the infecting recombinant *S. mutans* strain is preferably an auxotroph. An “auxotroph” is a bacterium that requires a source of a specific organic substance (s), in addition to a source of carbon in order to grow. For example, a “D-alanine auxotroph” is a bacterium that cannot grow without a source of D-alanine. Although auxotrophs can often persist (i.e. survive without growth) for a short period in the absence of the required organic substance, maintenance of auxotrophic colonization requires periodic supplements with that organic substance. For example, D-alanine auxotrophic bacteria require periodic D-alanine supplements in order to grow and maintain colonization of a niche, such as the oral cavity. Because mammals do not normally produce D-amino acids, and D-alanine is not normally secreted by organisms of the normal flora, D-alanine cannot be acquired by a D-alanine auxotroph from the normal milieu of the host’s oral cavity. Thus, the use of auxotrophs in the therapeutic method of the invention provides the advantage that oral colonization by the recombinant *S. mutans* can be interrupted by withholding the specific organic supplement, e.g. the D-alanine supplement.
  Bacterial auxotrophs can be generated using a variety of techniques well-known in the art, such as chemical mutagenesis, selection of spontaneous mutants, and/or recombinant techniques (e.g. transposon mutagenesis, replacement by recombination with a defective or nonfunctional gene). Preferably auxotrophic *S. mutans* strains are generated by either selection of spontaneous mutants, or by recombinant methods (e.g. introduction of a defect in a synthetic pathway for a D-amino acid). Preferably, D-alanine auxotrophic *S. mutans* strains are generated by introduction of a defect in the gene encoding alanine racemase, the enzyme that converts L-alanine to D-alanine.^5^
  Maintenance of oral cavity colonization by the auxotrophic, recombinant *S. mutans* strains of the invention can be achieved by oral administration of an auxotroph-maintaining amount of the organic substance for which the bacterium is auxotrophic. A “bacterial auxotroph-maintaining amount” is an amount of an organic substance sufficient to maintain viability of the bacterial auxotroph in the oral cavity. For example, where the recombinant *S. mutans* is auxotrophic for D-alanine, a “D-alanine bacterial auxotroph-maintaining amount” is an amount of D-alanine sufficient for survival of the D-alanine auxotrophic strain in the host’s oral cavity. In general, a single dose of a D-alanine bacterial auxotroph-maintaining amount of D-alanine contains from about 1 mg to 100 mg, preferably from about 5 mg to 75 mg, more preferably from about 10 mg to 50 mg, even more preferably from about 20 mg to 25 mg of D-alanine. The concentration of D-alanine in the pharmaceutical composition in the form of a solution ranges from about 0.01 mg/ml to 167 mg/ml (the latter being a saturated solution of D-alanine in water at 25°C.), preferably from about 0.1 mg/ml to 50 mg/ml, more preferably from about 1 mg/ml to 25 mg/ml. The concentrations of D-alanine in the pharmaceutical composition can vary according to the carrier used and the saturation point of D-alanine in that specific carrier.
  The organic substance required for maintenance of the auxotrophic, recombinant *S. mutans* in the oral cavity can be formulated in a variety of ways. For example, where the recombinant *S. mutans* is auxotrophic for D-alanine, the composition for maintenance of the D-alanine auxotroph can be formulated as a mouthwash, chewing gum, dental floss, toothpaste, chewable tablet, or any other formulation suitable for oral administration to the host’s oral cavity. In addition to the organic substance (e.g. D-alanine), the composition can additionally contain flavoring agents, coloring agents, fragrances, or other compounds, which increase the palatability of the composition and/or enhance patient compliance without compromising the effectiveness of the organic substance contained in the composition.
  The pharmaceutical compositions formulated for oral administration of the recombinant *S. mutans* strains of the invention and, where desired, their maintenance in the oral cavity must be developed within the intrinsic characteristics of the oral cavity and any desirable normal bacterial flora colonizing the oral cavity.^5^
  In case of *BCS3-L1,* designed as an effector strain for the prevention of dental caries, the most obvious potential adverse event is the acquisition of a wild-type LDH gene that would restore the ability of the cell to produce lactic acid. Such an event would initially occur in one cell among the millions that are present in the mouth. If a lactic acid producing revertant had a selective advantage over the effector strain, the ultimate result would be the outgrowth of a lactic acid producing strain equivalent to the one already present in the mouth of humans. For this reason, additional modifications were made to *BCS3-L1* to create *AJ2M* whose sole purpose is performing safety testing in human trails.^66^
  For human clinical trials, due caution suggested that additional genetic modifications of *BCS3-L1* were warranted in order to enable its rapid elimination from test subjects should an unexpected adverse effect manifest itself, and also to provide additional assurance that the strain has the maximum possible genetic stability. Thus, deletion mutations were introduced into the *DAL*-gene, which encodes alanine racemase necessary for endogenous synthesis of D-alanine. It was found that *AJ2M* was completely dependent on exogenous D-alanine, but could scavenge D-alanine from other plaque bacteria. As the human diet typically contains very little of this compound, it was felt that the *DAL* mutation would serve as a useful ‘recall’ mutation in the event of an unexpected adverse effect. Lowering of total bacterial load through daily application of chlorhexidine enabled virtually complete eradication of *AJ2M.* The addition of deletion in the *comE* gene provided additional safety as it had very low reversion frequency without, apparently, altering other important phenotypic properties of the effector strain. Thus, *AJ2M* appeared suitable for safe use in human clinical trials.^66^
2. Although mutants exhibiting defects in glucan synthesis have reduced cariogenicity in animal models, they are unlikely to compete successfully with glucan-synthesizing strains for prime plaque locations. ^67^ Specifically, the sucrose-dependent adherence of *S. mutans* to teeth ensures that bacteria will not be washed away with chewing or the flow of saliva, Interestingly, the rationale of the sucrose-dependent adherence could still be used for the effect strains to enhance their adherence ability, so as to let them occupy the same ecological niche in plaque as their more cariogenic counterparts.^27^ On the surface of S. mutans, there is a kind of GBL which could aggregate bacterium by binding the α-1, 6 glycosidic linkages. The adherence of *S. mutans* to teeth is the result of interaction between receptor and adhesion, the latter is a bacterium surface protein like component which could combine onto the complement and receptor of the tissue surface by specific stereochemistry way. So, GBL plays a very important role in the formation of dental plaque biomembrane and adherence of *S. mutans* itself. ^68^
  Sato, et al found that the gcrR gene acts as a negative transcriptional regular of bbpc gene which encoded *S. mutans* BGL. Amplification expression of bcrR gene restrained the gbpC gene, correspondingly resulted in decreased expression of gbpC and finally deprived the ability of *S. mutans* of sucrose dependent adherence. On the contrary, knockout of the gcrR gene resulted in overexpression of GBL. ^69^ So, the expression of GBL is regulated finely by the signaling system between bacterium and outside surrounding. Hence, Sun JH and others presumed that LDH-deficient *S. mutans,* which harbors an insertion deletion mutation in gcrR gene can result in overexpression of GBL and higher adherence to teeth than the wild-type *S. mutans* and will possess of both low-lower acid production and strong colonization potential. ^68^
3. Future work in the field of replacement therapy of dental caries may also take advantage of recent advances in the recombinant DNA technology and gene manipulation. The genes for various *S. mutans* virulence factors, such as LDH, bacteriocins, cell surface polysaccharides and adhesions could be cloned by modification of existing methods. The cloned genes could be modified, if need be, and used to construct an idealized effector strain by transformation^70^ or possibly conjugation^71^ into a host strain of *S. mutans.* Notwithstanding the public acceptance of such an approach, the potential use of recombinant DNA technology offers a degree of specificity and flexibility in the construction of effector strains that is not readily available with conventional genetic techniques.^16^

### SMaRT Technology (Trade Name for *Streptococcus Mutans:* Associated Replacement Therapy)^72^

It is a patented technology discovered by Oragenics’ Chief Scientific Officer Dr. Jeffrey Hillman.

SMaRT Replacement therapy is a single, painless topical treatment that has the potential to offer lifelong protection from the most tooth decay. The company had earlier been refused permission to test this replacement therapy in Phase I human trials because of fears of horizontal transmission of the genetically modified bacterium. Recently, permission has been granted to undertake a Phase I safety trial using an auxotrophic strain and denture wearing participants to determine the level of transmission of the bacterium. The company initiated its first Phase I trail with SMaRT Replacement strain *AJ2M* in the US in April 2005. The US Food and Drug Administration has given it a go ahead to conduct a second human safety trial (phase 1B) with this therapy. The trial will be conducted in compliance with the approved FDA protocol and designed to evaluate the safety and tolerability of the Company’s *SMaRT* Replacement Therapy(TM) in healthy, adult male subjects over a six-week period, with a long-term follow-up evaluation at six months. With this technology, a single 5-minute treatment is expected to provide lifelong protection against dental caries. As a result, *SMaRT* is anticipated to have a significant impact on the incidence of dental caries in human population. However, it is too early to determine the potential of this treatment method to prevent new carious lesions and arrest existing lesions without any significant adverse effects.

### About Oragenics

Oragenics, Inc. is a biopharmaceutical company with a pipeline of proprietary technologies. The company has a number of products in discovery, preclinical and clinical development with concentration in two main therapeutic areas, infectious disease and oncology. Our core pipeline includes products for use in the treatment of dental and periodontal infectious diseases, systemic bacterial infections, and weight loss. Oragenics will continue to build momentum toward licensing and commercialization of its lead products, SMaRT^TM^ replacement therapy, MU1140(TM) antibiotic, and Probiora3^TM^ probiotic by focusing its resources on these development programs and the initiation of clinical trials.” At the heart of breakthrough probiotic product is Oragenics’ patented Probiora3™ culture blend, a powerful proprietary ingredient that uses natural resident beneficial bacteria to crowd out problematic harmful bacteria while supporting healthy gums and teeth. At the same time, the continuous low doses of hydrogen peroxide produced naturally by the Probiora3™ blend of beneficial bacteria serves to whiten teeth.

## BENEFITS OF REPLACEMENT THERAPY^7^

A strong motivator for introduction of replacement therapy is the increased desire of consumers to use natural methods for health maintenance. Parenterally administered broad-spectrum antibiotics indiscriminately kill a wide variety of bacterial species associated with the host microflora resulting in formation of an ecological vacuum and encouraging superinfection and resistance development. By contrast, Bacteriocin-Like Inhibitory-Substance (BLIS) producing bacteria potentially offer a far more targeted solution to pathogen control. BLIS producers residing within the normal microflora are likely to cause little collateral killing of unrelated bacteria because they deliver narrow spectrum antimicrobial activity in concentrations that are probably inhibitory only to target bacteria in their immediate vicinity.

Modulation of the microflora composition by specific introduction of strains of ‘naturally occurring’ species that are capable of excluding colonization and/or infection by target pathogens could be viewed as the controlled manipulation of a process that otherwise occurs haphazardly in nature. Directed implantation of relatively harmless effector bacteria known to be strongly competitive with potential pathogens offers a cost-effective, long-term means of achieving tailor made protection for the host against specific bacterial infections. It might also foster increased herd protection through natural transmission of the effector strain to close contacts of the host. Furthermore, although consumer resistance might at present be an issue, genetic engineering ultimately presents a means of obtaining better-equipped effector strains, should equivalent natural isolates not be available.

## ISSUES STILL TO BE ADDRESSED

1. Strain *BCS3-L1* forms more plaque when grown *in vitro* in the presence of sucrose than strain *JH1140* does, probably reflecting a pH-related effect on glucosyl transferase activity.^27^ However, small increases in glucan formation *in situ* are not anticipated to result in significantly more plaque formation or plaque associated disease (e.g. gingivitis).^7^
2. Although reversion to LDH production (resulting in a strongly competitive cariogenic strain) following natural transformation of the effector strain is considered unlikely, it nevertheless constitutes a potential objection to implantation of LDH mutants. Further engineering of strain *BSC3-L1* (deletion of comE) is currently being undertaken to cripple its transformation capability. ^6^
3. The toxicity of mutacin 1140 has not yet been directly tested but the molecule is broadly similar to the lantibiotic nisin, which has been widely used as a food preservative for decades. Furthermore, no treatment related lesions have been detected in the organs of rats colonized with mutacin 1140 producers. ^27^
4. The potent mutacin output and different fermentation profile of strain *BCS3-L1* could upset plaque ecology and result in the proliferation of organisms with pathogenic potential. Interestingly, however the initial colonization studies with mutacin 1140-producing strains indicated apparent high specific inhibitory activity of the mutacin *in situ.* Exclusion of other MS was reported, with little or no other detectable modification to the total composition and balance of the subjects’ plaque microbiota. ^25^ This high specificity contrasts dramatically with the broad activity spectrum of mutacin 1140 when tested *in vitro.* ^15^ These findings indicate that BLIS-producing bacteria do not necessarily eliminate all sensitive coinhabitants in complex ecosystems, such as dental plaque. Presumably the distinct microhabitats found within complex biofilms, and the differing physiological growth states of the inhabitants enable the individual members of heterogeneous populations to co-exist, despite incompatibilities they might display in more homogeneous environments, such as laboratory cultures.^7^
5. Given the level of caution about testing a genetically modified bacterium it begs the question, will consumers be willing to allow a mouth rinse containing genetically modified bacteria to be administered to their children to slow the development of caries, even if the technology is shown to be effective?

## DIFFICULTIES AND POSSIBLE RISKS^7^

The normal microflora in healthy humans displays remarkable quantitative and qualitative stability, a reflection of a finely tuned climax community of dynamically interacting microbes that limits invasion by any foreign microbe or overgrowth by a minority member of the population. However, the equilibrium is regularly upset by various events, most dramatically by exposure to broad-spectrum antibiotics or antiseptics but also possibly following substantial nutritional, hormonal or physical changes to the microenvironment. Significant reductions in the numbers of individual components of the balanced microflora could result in overgrowth (superinfection) by previously suppressed minority members of the population. Similarly, the high intrinsic stability of the indigenous microbiota can present a major obstacle to modifying its composition by introduction of specific effector strains. Success is unlikely unless the effector strain is strongly competitive. Alternatively, the effector strain could be administered either before establishment of the microbial climax community (in the perinatal period) or upon creation of an appropriate niche following disruption of the microflora by exposure to antimicrobials.

Long-term retention of antibiotic producing effector strains might not be easily achieved. The additional energy and nutritional demands of antibiotic production could be sufficiently disadvantageous to organisms in some ecosystems that they will counterbalance any competitive benefit conferred by antibiosis. Under such circumstances, the antibiotic producing strain will gradually be replaced within the population. The selection of pathogens resistant to the effector strain remains a problem, particularly if microbial interference is largely mediated by antibiosis. Typically, however once an antibiotic selective pressure is removed resistant variants tend to be disadvantaged and are lost to the population. Also, the effector strain, no matter how harmless, could potentially initiate disease under unusual circumstances, such as immunosuppression, immunodeficiency, burns, drugs, stress and climatic variation. Because not all risks can be predicted, new opportunistic infections could conceivably be initiated. However, it seems the risks of this occurring would be minimized by the application of naturally occurring strains commonly isolated from balanced ecosystems in normal individuals.

## CONCLUSION

In general, replacement therapy employs a carefully constructed effector strain that provides a number of advantages over conventional prevention strategies and oral vaccines. For prevention of dental caries, a single colonization regimen that leads to persistent colonization by the effector strain should provide lifelong protection. In the event that the effector strain does not persist indefinitely in some subjects, reapplication can be done as the need arises without any significant added concern for safety or effectiveness. One of the greatest advantages of replacement therapy is that there is a minimal need for patient compliance relative to caries prevention although oral hygiene measures to prevent periodontal diseases will still be required. If ultimately successful, the use of genetic engineering to tailor an effector strain for replacement therapy for dental caries will encourage similar efforts to prevent other infectious diseases as well.
